# In Silico Exploration of CD200 as a Therapeutic Target for COVID-19

**DOI:** 10.3390/microorganisms12061185

**Published:** 2024-06-12

**Authors:** Vladimir Perovic, Sanja Glisic, Milena Veljkovic, Slobodan Paessler, Veljko Veljkovic

**Affiliations:** 1 Laboratory for Bioinformatics and Computational Chemistry, Institute of Nuclear Sciences VINCA, University of Belgrade, 11001 Belgrade, Serbia; sanja@vinca.rs; 2 Department of Clinical Laboratory Medicine, Hospital for Cerebrovascular Diseases Sveti Sava, 11000 Belgrade, Serbia; 3 Department of Pathology, University of Texas Medical Branch, Galveston, TX 77555, USA; 4 Biomed Protection, Galveston, TX 77550, USA

**Keywords:** COVID-19, SARS-CoV-2, variants, vaccine, therapeutic target, CD200

## Abstract

SARS-CoV-2, the pathogen causing COVID-19, continues to pose a significant threat to public health and has had major economic implications. Developing safe and effective vaccines and therapies offers a path forward for overcoming the COVID-19 pandemic. The presented study, performed by using the informational spectrum method (ISM), representing an electronic biology-based tool for analysis of protein–protein interactions, identified the highly conserved region of spike protein (SP) from SARS-CoV-2 virus, which is essential for recognition and targeting between the virus and its protein interactors on the target cells. This domain is suggested as a promising target for the drug therapy and vaccines, which could be effective against all currently circulating variants of SARS-CoV-2 viruses. The analysis of the virus/host interaction, performed by the ISM, also revealed OX-2 membrane glycoprotein (CD200) as a possible interactor of SP, which could serve as a novel therapeutic target for COVID-19 disease.

## 1. Introduction

Since the emergence of COVID-19, caused by the novel coronavirus SARS-CoV-2, more than 200 million people have been infected, and more than 4.5 million have died from this disease [1]. The COVID-19 pandemic’s effects extend beyond immediate health concerns, significantly disrupting global socio-economic systems. These extensive disruptions have exacerbated existing disparities both within and between nations, further straining healthcare systems [2]. The immense burden of treating infected individuals has stretched healthcare resources to their limits, diverting attention and resources away from other critical medical needs. Furthermore, it has had enduring impacts on attitudes toward long-term health and techniques for controlling chronic diseases, such as cardiovascular diseases [3]. The pandemic has exposed significant gaps in healthcare systems and everyday medical care, requiring immediate action. An analysis of different countries’ responses underscores the pressing necessity to overhaul healthcare systems, which involves using telemedicine and mobile health technologies to ensure continuous patient care and minimize the risk of contagion [4]. These challenges underscore the critical need for effective therapeutic interventions. They are exacerbated by the SARS-CoV-2 virus’s ability to undergo continual mutation, leading to novel genetic variations that circulate globally throughout the COVID-19 pandemic [5,6]. Some of these mutations are linked to alterations in receptor binding, facilitating easier transmission from person to person, decreased neutralization by antibodies produced from prior infection or vaccination, and reduced effectiveness of treatments [5,6,7,8]. As the virus undergoes further changes, these mutations provide substantial difficulties for current treatment strategies, requiring continuous adjustment and advancement of therapeutic approaches. Omicron subvariants continue to evolve rapidly under the pressure of humoral immunity developed through vaccination and infection. These novel subvariants significantly decrease the efficiency of existing antibody treatments as well as the humoral immunity established through vaccination and infection [9]. The growing number of Omicron subvariants shows how important it is to find new conservative antigenic epitopes. These can help make broad-spectrum, strong neutralizing antibodies and come up with better ways to vaccinate against future variants. This trend emphasizes the need for novel ways of detecting and monitoring viral mutations in real time. Recent advancements in genomic sequencing and bioinformatics have proven pivotal in identifying and tracking these mutations, facilitating a more agile response to the COVID-19. The study by Bhat et al. (2021) [10] has highlighted how genomic surveillance can inform public health decisions and guide the development of targeted interventions. Such capabilities are critical not only for managing COVID-19 but also for preparing for future pandemics, underscoring the need for global cooperation in health technology and data sharing [10].

To address the continued problems posed by SARS-CoV-2 and its developing variants, it is critical to identify new treatment targets that can provide wide protection against both current and future strains. These approaches should not only attempt to boost the immune response but also keep the virus from evading immune identification. SARS-CoV-2, similar to other viruses, has evolved intricate strategies to evade the human immune system, enabling it to reproduce and endure [11]. Understanding how SARS-CoV-2 evades the body’s natural immune defenses is critical for appreciating its potential to cause disease and developing novel treatment strategies [11]. Identifying viral proteins responsible for immune evasion can facilitate the development of novel antiviral treatments targeting these proteins.

We analyzed subunit 1 of the spike protein (SP1) from different SARS-CoV-2 variants to determine some conserved properties of these viruses, which could serve as a base for developing the universal vaccine and therapy for COVID-19. In our analysis, we used the bioinformatics tool based on the electron–ion interaction potential (EIIP), the informational spectrum method (ISM) [12]. The EIIP is the molecular descriptor that determines the long-range properties of biological molecules, which are essential for their long-distance (up to 1000 Å) interactions [12]. These interactions allow for the specific recognition and targeting of interacting molecules in the biological space, which many other molecules occupy (e.g., the mammalian cell contains 2–4 billion molecules). Previously, the ISM was used to analyze the conserved properties of HIV, Ebola virus, and influenza viruses [13,14,15]. Of note is that this bioinformatics tool was also used in the first ever published molecular analysis of SARS-CoV-2, suggesting (i) the angiotensin-converting enzyme 2 (ACE2) as the natural receptor of this virus and (ii) the cell-to-cell transmission of the COVID-19 virus [16].

The presented study revealed the highly conserved domain of SARS-CoV-2 SP1, which is essential for recognizing and targeting the virus and receptor(s) on the target cells. This domain represents a promising target for drug therapy and vaccines, which could be effective against all currently circulating variants of COVID-19 viruses. This analysis also suggests that OX-2 membrane glycoprotein (CD200) is a possible novel interactor of SP1.

## 2. Materials and Methods

### 2.1. Viruses

We analyzed the subunit 1 of S protein (SP1) from following SARS-CoV-2 variants (GISAID database [17,18]):

alpha (hCoV-19/Japan/YCH0355/2021)

beta (hCoV-19/Japan/TY9-676-P0/2021)

gamma (hCoV-19/Japan/TY9-675-P0/2021)

delta (hCoV-19/Romania/B-16596/2021)

epsilon (hCoV-19/USA/AR-UNM-UAMS83MD132/2021)

zeta (hCoV-19/USA/ME-HETL-J3758/2021)

eta (hCoV-19/Benin/315465/2021)

theta (hCoV-19/USA/NJ-CDC-LC0084533/2021)

lota (hCoV-19/USA/FL-TGH-0865/2021)

kappa (hCoV-19/Ireland/CE-NVRL-Q23IRL85883/2021)

lambda (hCoV-19/Chile/AT-119048/2021)

The SARS-CoV-2 S protein reference sequence (YP_009724390) is used in the analysis as the wild type (WT).

### 2.2. Informational Spectrum Method

Previously, the two-step model of molecular interactions in biological systems was proposed [12,19]. The first step is recognition and targeting between interacting molecules, and the second is chemical binding. The first step, which determines the number of productive collisions between interacting molecules, is characterized by the selective attractive forces acting on the distances between 5 and 1000 Å. The second step is the chemical binding between molecules based on the electrostatic forces acting on the distances <5 Å. Both steps represent “required and sufficient” conditions for efficient interaction between biological molecules. Notably, the selective long-distance attractive electromagnetic forces (up to 1000 Å) between biological molecules have recently been experimentally confirmed [20].

It has been proposed that the number of valence electrons (AQVN) and the electron-ion interaction potential (EIIP), representing the main energy term of the valence electrons, are essential physical parameters of biological molecules determining their long-range properties. These molecular descriptors are derived from the “general model pseudopotential” [19,21,22] and are defined by the following equations:

W = 0.25Z* sin(1.04π Z*)/2π,(1)

where Z* is the AQVN determined by

Z* = ∑_i=1_…_m_ n_i_Z_i_/N (2)

where Z_i_ is the valence number of the i-th atomic component, n_i_ is the number of atoms of the i-th component, m is the number of atomic components in the molecule and N is the total number of atoms. The EIIP values calculated according to Equations (1) and (2) are in Rydbergs (Ry = 13.5 eV).

The molecular descriptors AQVN and EIIP served as a base for the development of the informational spectrum method (ISM), representing the virtual spectroscopy method for the analysis of proteins and DNA/RNA molecules. The physical and mathematical basis of ISM is described in detail elsewhere (for review, see refs. [19,20,21,22]), and here, the method will be presented only briefly. First, the primary structure of a protein is transformed into a numerical sequence by the representation of each amino acid by the corresponding value of the electron–ion interaction potential (EIIP) (Table 1). The obtained numerical sequence is then subjected to discrete Fourier transformation, defined as follows:

X(n) = ∑_m=1_…_n_ x(m)e^−i2πnm/N^, n = 1…N/2 (3)

where x(m) is the m-the member of a given numerical series, N is the total number of points in this series and X(n) are discrete Fourier transformation (DFT) coefficients. These coefficients describe the amplitude, phase and frequency of sinusoids, which comprise the original signal. The absolute value of complex Fourier transformation defines the amplitude and phase spectrum. Both spectral functions contain all the information regarding the original sequence. However, when it comes to protein analysis, the pertinent information is displayed in the energy density spectrum, which is described as follows:

S(n) = X(n)X*(n) = |X(n)|^2^, n = 1…N/2 (4)

In this way, sequences are analyzed as discrete signals. Their points are assumed to be equidistant, and the distance is set at an arbitrary value of d = 1. Then, the maximum frequency in the spectrum is F = 1/2d = 0.5. The total number of points in the sequence influences the resolution of the spectrum only. The resolution of the N-point sequence is 1/N. The n-th point in the spectral function corresponds to the frequency f = n/N. Thus, the initial information defined by the sequence of amino acids can now be presented as an informational spectrum (IS), representing the series of frequencies and corresponding amplitudes.

The primary structures of interacting proteins or proteins interacting with a common interactor encode the common information represented by the same code/frequency pair(s) in their IS. Cross-spectrum or consensus informational spectrum (CIS) determines this common informational characteristic of sequences. The following equation obtains the CIS of two spectra:

C(j) = ∏_i=1_…_M_S(i,j), j = 1…N/2 (5)

S(i,j) is the jth element of the i-th power spectrum, and C(j) is the j-th element of CIS. Peak frequencies in CIS represent common information and are characterized by the amplitude and the signal-to-noise ratio (S/N, ratio of the amplitude value on a particular frequency and the sum of amplitudes on all frequencies in IS).

## 3. Results

**Figure 1:**
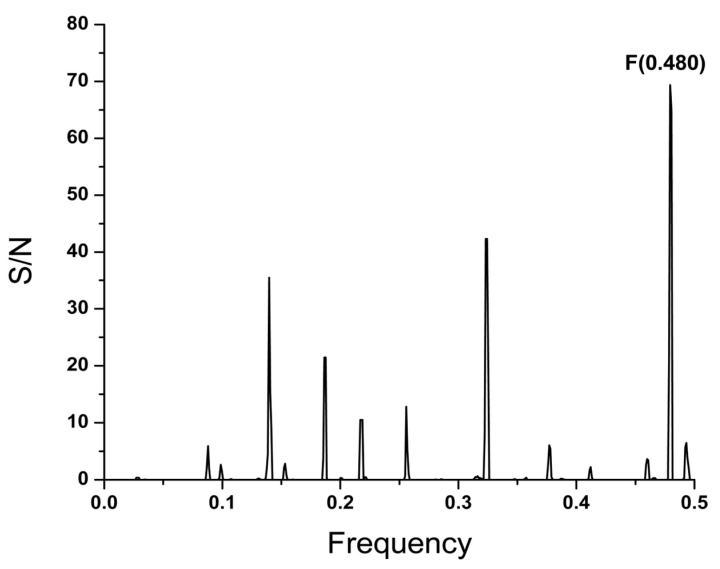
The cross-spectrum of the SP1 protein from alpha, beta, gamma, delta, epsilon, zeta, eta, theta, iota, kappa and lambda variants of SARS-CoV-2 virus.

In order to identify the conserved information in different SARS-CoV-2 virus variants, the cross-spectrum of the SP1 protein from alpha, beta, gamma, delta, epsilon, zeta, eta, theta, iota, kappa, and lambda SARS-CoV-2 viruses was calculated. The common information encoded in SP1 from analyzed viruses is represented by the dominant peak on the frequency F(0.480) in the cross-spectrum presented in Figure 1. It has been previously shown that the dominant frequency in the informational spectrum of viral envelope proteins corresponds to the interaction between the virus and its receptor [15,16]. Accordingly, the information represented with the frequency F(0.480) determines the long-range interaction between SP1 and the homing protein(s) (receptor/co-receptor) on the surface of the target host cell.

**Figure 2:**
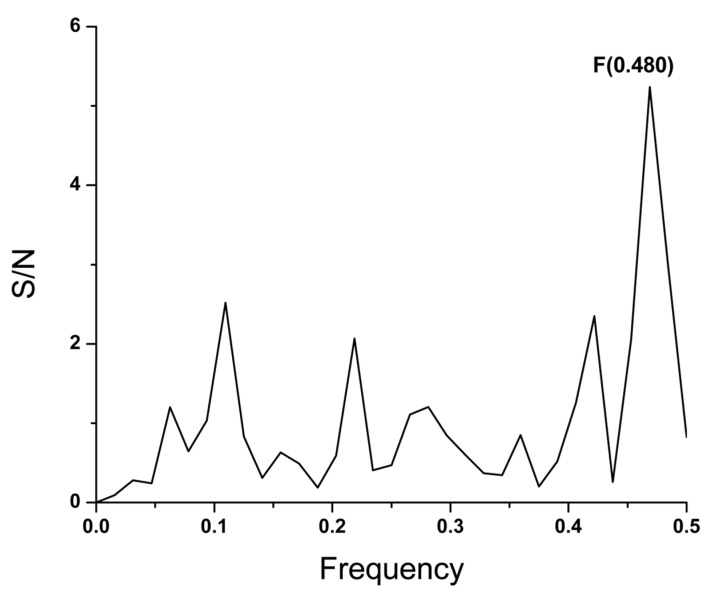
The informational spectrum of the BmP domain of the SP1 protein from SARS-CoV-2 virus (hCoV-19/Wuhan/Hu-1/2019).

**Figure 3:**
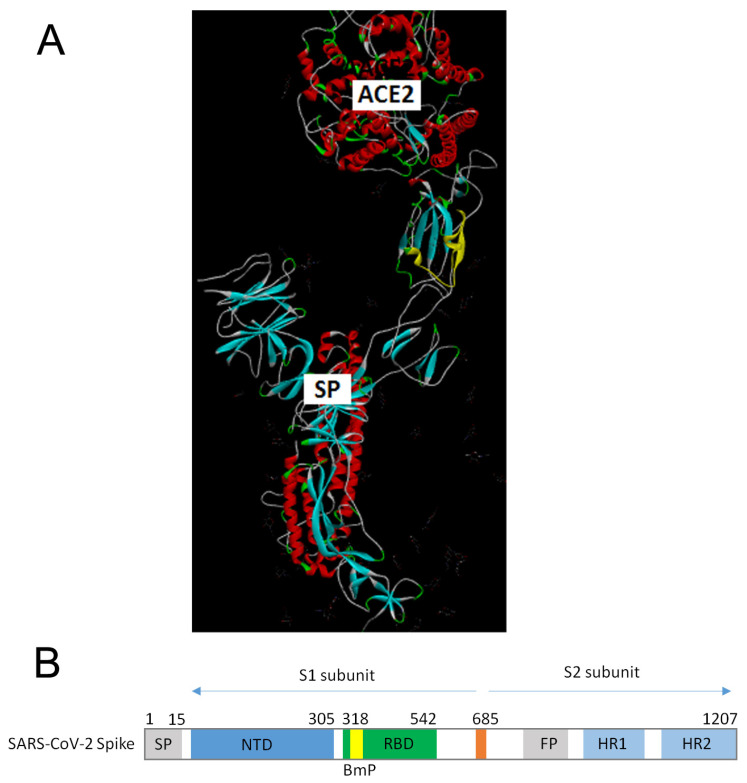
The BmP domain in SP protein from SARS-CoV-2 virus. (**A**) The position of BmP (yellow) in the complex of SP protein and the ACE2 receptor. (**B**) Schematic presentation of the BmP position in SP protein. (SP: signal peptide, RBD: Receptor Binding Domain, 685: S1–S2 boundary (positions 685–686), FP: Fusion Peptide, HR1: Heptad Repeat 1, HR2: Heptad Repeat 2).

The SP1 amino acid sequence from the SARS-CoV-2 was scanned to look for the domain with the highest contribution to the information represented by the frequency F(0.480). This analysis revealed that domain 335–367 (further denoted BmP) is essential for recognizing and targeting SARS-CoV-2 SP1 and its protein interactor on the target host cell (Figure 2). The Bmp is located in the N-terminus of the receptor binding domain (RBD) of SP1 (Figure 3A). As can be seen in Figure 3B, BmP is close to the domain of SP1, participating in its chemical binding to the ACE2 receptor. 

**Figure 4:**
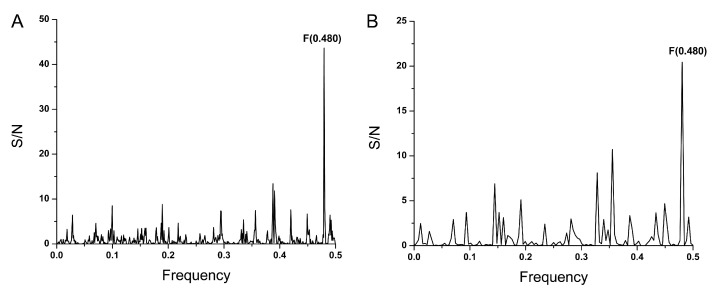
(**A**) The cross-spectrum between SP1 protein from SARS-CoV-2 virus (hCoV-19/Wuhan/Hu-1/2019) and the receptor CD200R2. (**B**) The cross-spectrum between the CD200 (OX-2) and the receptor CD200R2.

The UniProt database release 2024_01 [23] was screened by ISM for human proteins with the dominant peak on the frequency F(0.480) in the cross-spectrum with SP1. First, the UniProt database was preselected, and reviewed proteins (Swiss-Prot) were extracted based on the criteria plasma membrane, cell surface, and Homo sapiens. This analysis used SP1 from the prototype virus hCoV-19/Wuhan/Hu-1/2019. This analysis showed that CD200 (OX-2) has the highest peak on F(0.480) in cross-spectrum with SP1 (Figure 4A). The calculation of the cross-spectra between CD200 and its receptors showed that the frequency F(0.480) characterizes the CD200/CD200R2 interaction (Figure 4B), suggesting that this frequency is also important for this interaction. These results indicate that CD200 could represent a possible host interactor of SARS-CoV-2 SP1, or SARS-CoV-2 SP1 could act as the CD200 mimetic that may modulate the CD200:CD200R pathway.

**Figure 5:**
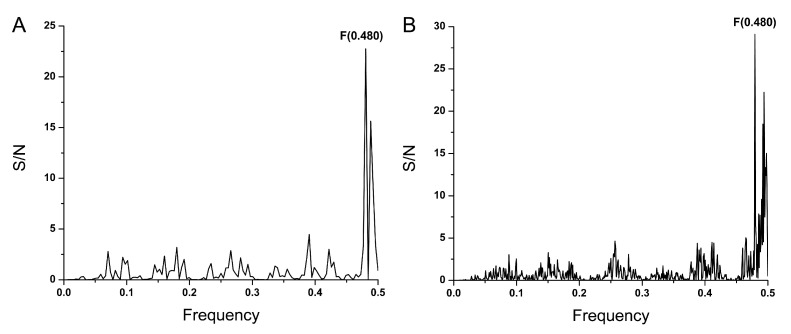
(**A**) The cross-spectrum between peptide VTWQKKKAVSPANM and the receptor CD200R2. (**B**) The cross-spectrum between peptide VTWQKKKAVSPANM and SP1 protein from SARS-CoV-2 virus (hCoV-19/Wuhan/Hu-1/2019).

It has been shown previously that peptide VTWQKKKAVSPANM is an effective inhibitor of the interactions between CD200 and its receptors [24]. The peak with the highest amplitude in the cross-spectrum between this peptide and CD200R2 is on F(0.480) (Figure 5A). The dominant peak in the cross-spectrum of SP1 and peptide VTWQKKKAVSPANM is also on the same frequency (Figure 5B), suggesting that this viral protein could modulate the CD200/CD200R interaction.

## 4. Discussion

SARS-CoV-2, like other coronaviruses, stands out among RNA viruses because of their vast genomes (~30 kb) associated with low mutation rates. To achieve this accuracy, the COVID-19 virus has acquired nsp14, a bifunctional enzyme able to methylate the viral RNA cap and excise erroneous mutagenic nucleotides inserted by the nsp12 enzyme. Despite this ability to control mutations [25], of 1,587,509 viruses submitted from January to May 2021 to the GISAID database [17], 1391 have SP1 with the unique combination of mutations. According to the criteria, which include specific genetic markers that have been associated with changes to receptor binding, reduced neutralization by antibodies generated against previous infection or vaccination, reduced efficacy of treatments, potential diagnostic impact, or predicted increase in transmissibility or disease severity, mutants are divided into 11 groups which are defined as the variants of COVID-19 viruses [26]. These variants have different effects on the efficacy of COVID-19 vaccines. With the original alpha variant, breakthrough infections were exceedingly rare, and vaccinated people were not found with high viral loads, meaning they almost never transmitted the virus to unvaccinated people. The data recently published by the CDC showed that fully vaccinated people who get infected with the delta variant can spread the virus because they carry as much of the virus in their noses as unvaccinated people [27].

Developing a universal vaccine or therapeutic approach could save lives and reduce strain on healthcare infrastructure, allowing for a more sustainable response to COVID-19 and future outbreaks [18]. The critical problem in developing a universal COVID-19 vaccine is ensuring it is effective against all existing and future SARS-CoV-2 variants. This endeavor is complex [28], as illustrated by decades of research into developing a universal influenza vaccine, which has had minimal success [29]. The main obstacle to influenza is its genetic diversity. Although SARS-CoV-2 mutates more slowly than influenza, the constant emergence of new variants threatens the efficacy of existing COVID-19 vaccines. A universal vaccine must account for potential future lineages and variants whose characteristics are currently unknown. Consequently, it is it is not feasible to assess the efficacy of universal vaccines in clinical trials that evaluate the incidence of illness or infection with the target viruses following immunization.

This study introduces a novel approach, a bioinformatics analysis of the SARS-CoV-2 SP1 protein, which offers potential solutions to some of the challenges in developing a universal COVID-19 vaccine. Previous research using the same informational spectrum methodology has shown that hemagglutinin from different subtypes of influenza A viruses contains conserved information, represented by a common frequency component in their informational spectra [30,31,32,33]. This information was used to design antigens for vaccines that were effective against various influenza viruses [30,31,32,33].

As illustrated in Figure 1, the informational cross-spectrum of SP1 in all currently circulating SARS-CoV-2 variants is characterized by the common frequency component F(0.480). According to the informational spectrum method (ISM) concept, this frequency corresponds to the long-distance interaction of these viruses with a common receptor or co-receptor on target cells. The domain BmP, which significantly contributes to the information represented by F(0.480), is highly conserved in SP1 across all 11 variants and is located near the region of SP1 involved in chemical binding to the ACE2 receptor, suggesting that BmP could be a promising universal therapeutic and vaccine target for all variants of the COVID-19 virus. Notably, it was shown that cross-reactive antibodies induced by common cold coronaviruses that bind to the epitope in the BmP domain provide some degree of protective immunity against recent emerging SARS-CoV-2 variants [34]. This underscores the importance of the highly conserved domain of SARS-CoV-2 SP1 in recognizing and targeting the virus to receptors, thereby validating the in silico approach. Screening 16,766,406 viruses in the GISAID database (accessed on 2 June 2024) [18], which includes sequences of Omicron and its current variants of interest (VOIs), revealed only two rare mutations (G339H, R346T) within BmP, further supporting its potential as a universal target. The ISM analysis of virus–host interactions also identified the OX-2 membrane glycoprotein (CD200) as a potential interactor of the SP protein. This discovery suggests CD200 could serve as a novel therapeutic target for COVID-19.

Even with a promising target identified, delivering a therapeutic agent to a target effectively remains a challenge in nanomedicine. One of the most significant issues is the efficient delivery of nanodrugs to therapeutic targets [35]. Developing effective targeting ligands that specifically accumulate at the target site rather than in other tissues is a significant challenge. Using the informational spectrum method, a concept of long-range intermolecular interactions for recognition and targeting between drugs and therapeutic targets has been shown to aid in designing targeting peptides for delivering nanodrugs to therapeutic targets [35]. The presented results suggest that BmP and other peptides encoding information corresponding to F(0.480) could be used as targeting peptides for nanodrugs to treat COVID-19.

The presented results of the bioinformatics analysis in this study revealed the protein CD200 as a possible host interactor of the SARS-CoV-2 SP1 protein. The SP1/CD200 interaction could play a role in the COVID-19 pathogenesis. Previously, it was suggested that CD200 plays a critical role in lung immune homeostasis and the severity of influenza infection. Snelgrove and co-workers showed that mice lacking CD200 had more macrophage activity and enhanced sensitivity to influenza infection, leading to delayed inflammation resolution and, ultimately, death [36]. The administration of CD200 agonists that bind CD200R prevented inflammatory lung disease [36]. Many viruses have acquired functional homologs of CD200, implying that viral exploitation of this pathway is evolutionary advantageous [37]. Research on viral CD200 (vCD200) suggests that it functions as a viral homolog of human CD200, particularly in herpesviruses like rhesus rhadinovirus (RRV) and Kaposi’s sarcoma-associated herpesvirus. RRV vCD200 affects immunological responses by lowering tumor necrosis factor levels in macrophages, which aids viral survival and contributes to immune evasion [38]. These findings highlight the strategic role of vCD200 in herpesviruses as a mimic of the host’s CD200, suppressing immune responses and increasing viral persistence and pathogenicity. The results from the studies emphasize how herpesviruses, particularly HHV-8 and CMV, employ vCD200 to avoid immune detection and establish long-term infections. Mouse cytomegalovirus uses the CD200 pathway to survive in mucosal tissue by expressing the inhibitory ligand CD200 [39]. These changes are critical for viruses to remain in the host because they modulate immune responses and decrease antiviral activity [40].

Studies have shown that CD200 interacts with receptors other than the well-known CD200R1. The CD200-CD200R2 interaction is rapidly becoming recognized for its function in manipulating the host immune system by viral infections. Viral pathogens, such as herpesviruses, can express CD200 orthologues that bind to host CD200 receptors, including CD200R2, to suppress immune responses and facilitate viral persistence [41]. This engagement modulates dendritic cell (DC) functions, altering cytokine production and immune activation to create an anti-inflammatory environment that supports viral replication [42]. Additionally, CD200R2 interaction promotes the development of regulatory T cells (Tregs), which further suppresses antiviral immune responses and aids in immune evasion [42]. By exploiting these mechanisms, viruses can enhance their persistence within the host [37]. These insights into CD200-CD200R2 signaling highlight its complexity and potential as a target for therapeutic interventions to boost antiviral immunity and control chronic infections.

SARS-CoV-2 may use immune modulation pathways such as the CD200-CD200R pathway to suppress host immunological responses and promote viral survival and proliferation. This route could play an important part in SARS-CoV-2’s immune evasion mechanisms, making it a potential therapeutic target [43]. The interaction between CD200 and its receptor CD200R is known to suppress immune responses by inhibiting the PI3K/Akt signaling pathway [44], which SARS-CoV-2 may also use to weaken host immune defenses and facilitate viral persistence in the respiratory tract [44]. SARS-CoV-2 infects via the respiratory system and causes an inflammatory response by affecting a variety of cell types, including type II alveolar epithelial cells [44]. The SARS-CoV-2 Spike (S) protein has been linked to the pathogenesis of COVID-19. During the early stages of infection, the S protein inhibits inflammatory responses in alveolar epithelial type II cells by activating the PI3K/Akt pathway [45]. If the S protein influences the CD200/CD200R interaction, it could indirectly affect the PI3K/AKT pathway by altering immune responses through CD200R signaling which would be consistent with our results.

Previous studies have shown that peptides can effectively modulate CD200- CD200R interactions [46,47]. These peptides can act as agonists or antagonists of CD200:CD200R interactions, thus modulating immunological responses. The ability to either activate or inhibit this pathway suggests that modulation of CD200:CD200R interactions has therapeutic potential. This approach may have significant implications for various human disorders, including viral infections, by enhancing immune responses or promoting immunological tolerance. Understanding and harnessing these interactions can lead to novel therapeutics that improve immune modulation and disease outcomes [47]. 

In this study, the ISM, an electronic biology-based tool for analyzing protein–protein interactions, was used to identify the highly conserved region of the spike protein (SP) from the SARS-CoV-2 virus. This region is crucial for the virus’s recognition and targeting of protein interactors on target cells. It represents a promising target for drug therapy and vaccines effective against all currently circulating SARS-CoV-2 variants. Additionally, the ISM analysis of virus–host interactions revealed the OX-2 membrane glycoprotein (CD200) as a potential interactor with SP, suggesting CD200 could serve as a novel therapeutic target for COVID-19. Previously, the ISM has been successfully applied and experimentally validated in various areas of protein research, including structure-function analysis of protein sequences, prediction of new protein interactors, and identification of protein domains responsible for long-range interactions. ISM has identified functional protein domains that serve as candidate therapeutic targets for drugs against several pathogenic diseases, including HIV-1, anthrax, and influenza [48]. Additionally, ISM has accurately predicted protein–protein interactions confirmed through experimental methods. For instance, the direct interaction between the β2-adrenergic receptor (β2AR) and the insulin receptor (IR) was initially identified using bioinformatics and later confirmed through bioluminescence resonance energy transfer (BRET) [49]. Furthermore, the design and optimization of nanobody-derived peptides that interact with the β2-adrenergic receptor by ISM were experimentally validated [50], demonstrating the effectiveness of the ISM approach in guiding experimental validation and therapeutic development. 

In this study, we selected interactor candidates based on the best signal-to-noise ratio and amplitude ratio in the cross-spectrum between SP1 and the interactor candidate. The Supplementary Table S1 shows the remaining 13 of the total 15 selected interactors, with CD200 and CD200 receptor 2 being the primary focus of this manuscript. We also identified alpha integrins as potential targets during the in silico screening of the UniProt database. This finding aligns with the existing literature that reports about the involvement of various alpha integrins in SARS-CoV-2 infection. The study of Norris et al. [51] provided evidence that the receptor binding domain (RBD) of the spike protein from SARS-CoV-2 interacts with alpha integrins αvβ3 and αvβ6. This interaction has the potential to affect cellular adhesion and signaling cascades. In addition, it was demonstrated that alpha integrin α5β1 plays a crucial role in facilitating the fusion of cells and triggering inflammatory responses [52]. Also, in silico identified, Syndecan-4, prevalent in the lungs, promotes the transmission of the Delta variant of SARS-CoV-2 by binding to its altered spike glycoprotein. This enables cellular entry and displays a greater affinity for heparan sulfate proteoglycans than ACE2 [53]. 

The interaction of CD200 with its receptor contributes to creating an immune-suppressive tumor microenvironment. The methods developed in the invention aimed to reverse or modulate immune suppression in patients with diseases or disorders resulting from abnormal cell growth [24]. The patent demonstrated that the peptide VTWQKKKAVSPANM acts as a CD200R antagonist, effectively inhibiting the interactions between CD200 and its receptors. The use of antagonist peptides was proposed as a strategy to block the suppressive effects of CD200, thereby enhancing immunotherapy. This action can counteract the immune-suppressive effects mediated by the CD200/CD200R interaction. Interestingly, the dominant peak in the cross-spectrum of SARS-CoV-2 SP1 and peptide VTWQKKKAVSPANM occurs at the same frequency, suggesting that SARS-CoV-2 SP1 could modulate the CD200/CD200R interaction. Since SARS-CoV-2 can induce immune suppression, targeting CD200/CD200R interactions with antagonists could alleviate immune suppression in COVID-19 patients.

It has been reported that inhibiting the CD200-CD200R1 immune checkpoint system has favorable effects in battling coronaviruses, including the restoration of interferon (IFN) production and increased virus clearance [54,55]. Recently, it was shown that Jingfang granules can protect the lungs from acute injury and mitigate the recruitment and overactive alveolar macrophage-induced inflammation via the CD200-CD200R immunoregulatory signal axis. These results provide the basis for clinical applications of this traditional Chinese medicine formulation in treating COVID-19 [56]. These findings indicate that using CD200 inhibitors could counteract the immunosuppressive effects of the CD200-CD200R1 pathway, thereby restoring interferon production and increasing viral clearance. Consequently, CD200R antagonists may represent a promising therapeutic approach for treating COVID-19 and other coronavirus infections. If this hypothesis were experimentally confirmed, the SP1/CD200 interaction could be a novel therapeutic target for COVID-19 disease.

## 5. Conclusions

The presented results suggest (i) that the highly conserved BmP domain of SARS-CoV-2 SP1, which is important for recognition and targeting between virus and its receptor(s), could serve as a therapeutic target for drugs and as a base for the development of a universal vaccine and the targeting peptides for nanodrugs for treatment of COVID-19 disease, and (ii) that SP1 could modulate the interaction between the CD200 protein and CD200R, suggesting the CD200:CD200R pathway as a potential novel therapeutic target for COVID-19 disease.

## Figures and Tables

**Table 1:** The electron–ion interaction potential (EIIP) used to encode amino acids.

Amino Acid	EIIP [Ry]
Leu	0.0000
Ile	0.0000
Asn	0.0036
Gly	0.0050
Glu	0.0057
Val	0.0058
Pro	0.0198
His	0.0242
Lys	0.0371
Ala	0.0373
Tyr	0.0516
Trp	0.0548
Gln	0.0761
Met	0.0823
Ser	0.0829
Cys	0.0829
Thr	0.0941
Phe	0.0946
Arg	0.0959
Asp	0.1263

## Data Availability

The original contributions presented in the study are included in the article/Supplementary Material, further inquiries can be directed to the corresponding author.

## Supplementary Materials

The following supporting information can be downloaded at: https://www.mdpi.com/article/10.3390/microorganisms12061185/s1, Table S1: The additional 13 selected interactors of the total 15, excluding CD200 and CD200R2, with the best signal-to-noise ratio and amplitude ratio in the cross-spectrum with SARS-CoV-2 SP1 protein.
