# Supplementary material for: In Silico Exploration of CD200 as a Therapeutic Target for COVID-19
Source: Microorganisms. 2024 Jun 12;12(6):1185. doi: 10.3390/microorganisms12061185 (PMC11205781; doi:10.3390/microorganisms12061185)
Supplement: Supplementary file 1 [file microorganisms-12-01185-s001.zip › microorganisms-3043490-supplementary.pdf]

Supplementary Table S1

The additional 13 selected interactors of the total 15, excluding CD200 and CD200R2, with the best signal-to-noise ratio and amplitude ratio in the cross-spectrum with SARS-CoV-2 SP1 protein.

**Protein**	**UniProt ID**
Integrin alpha-4 OS	P13612
Integrin alpha-D	Q13349
Cadherin-5 OS	P33151
Inactive tyrosine-protein kinase transmembrane receptor ROR1	Q01973
High affinity cationic amino acid transporter 1	P30825
Glypican-2 OS=Homo sapiens	Q8N158
Mucin-13 OS=Homo sapiens	Q9H3R2
Arrestin domain-containing protein 3	Q96B67
Mannose-binding protein C	P11226
Free fatty acid receptor 1 OS	O14842
Protein S100-A9	P06702
Hematopoietic cell signal transducer	Q9UBK5
Syndecan-4	P31431
